# Supplementary material for: Pyramiding of gn1a, gs3, and ipa1 Exhibits Complementary and Additive Effects on Rice Yield
Source: Int J Mol Sci. 2022 Oct 18;23(20):12478. doi: 10.3390/ijms232012478 (PMC9604080; doi:10.3390/ijms232012478)
Supplement: Supplementary file 1 [file ijms-23-12478-s001.zip › ijms-1939113-supplementary figures.pdf]

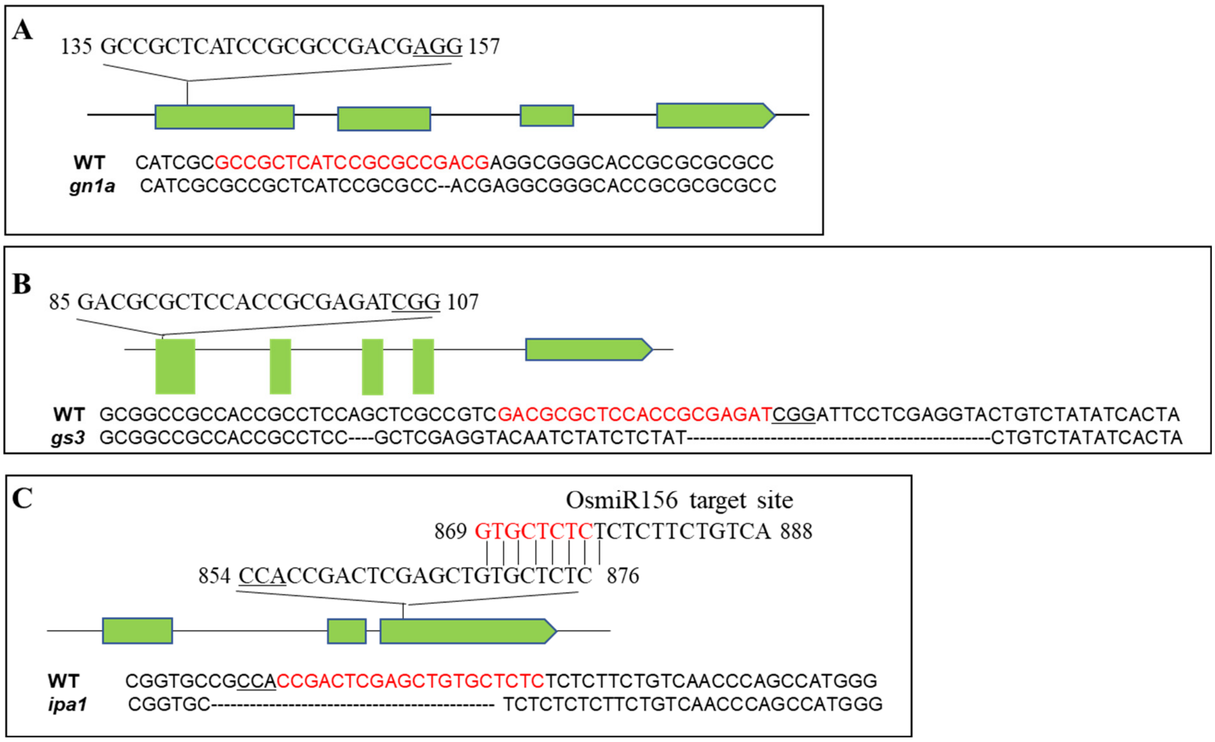

Figure S1. Generation of null alleles of *gn1a*, *gs3*, and *ipa1* using CRISPR/Cas9 gene-editing technology. (A) Schematic description of the CRISPR target and the mutant of *gn1a* generated, the gn1a mutation contains a 1-bp deletion in the *gn1a* coding region. (B) Schematic description of the CRISPR target and the mutant of *gs3* generated, the *gs3* mutation contains a 47-bp deletion and 24 insertion in the *gs3* coding region. (C) Schematic description of the CRISPR target and the mutant of ipa1 generated, the ipa1 mutation contains a 21-bp deletion in the ipa1 coding region and this deletion region is also the target site of OsmiR156. The underlined area is the PAM site of the target sequence. The alignment of the WT and mutant sequence were shown on the figure.

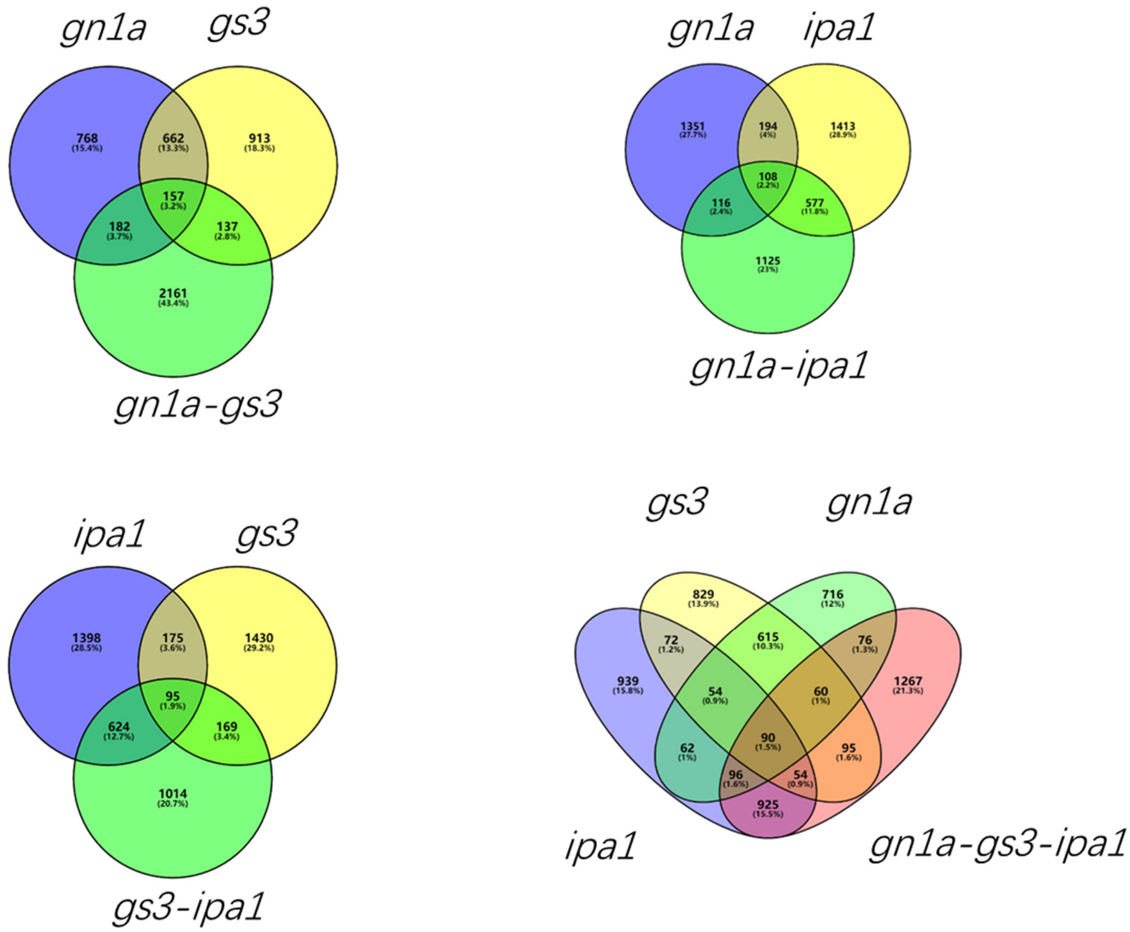

Figure S2. Venn diagram showing the overlap of the DEGs groups from 4 pyramided lines.
