# Supplementary material for: Pyramiding of gn1a, gs3, and ipa1 Exhibits Complementary and Additive Effects on Rice Yield
Source: Int J Mol Sci. 2022 Oct 18;23(20):12478. doi: 10.3390/ijms232012478 (PMC9604080; doi:10.3390/ijms232012478)
Supplement: Supplementary file 1 [file ijms-23-12478-s001.zip › Fig. S1,S2.pptx]

## Slide 1
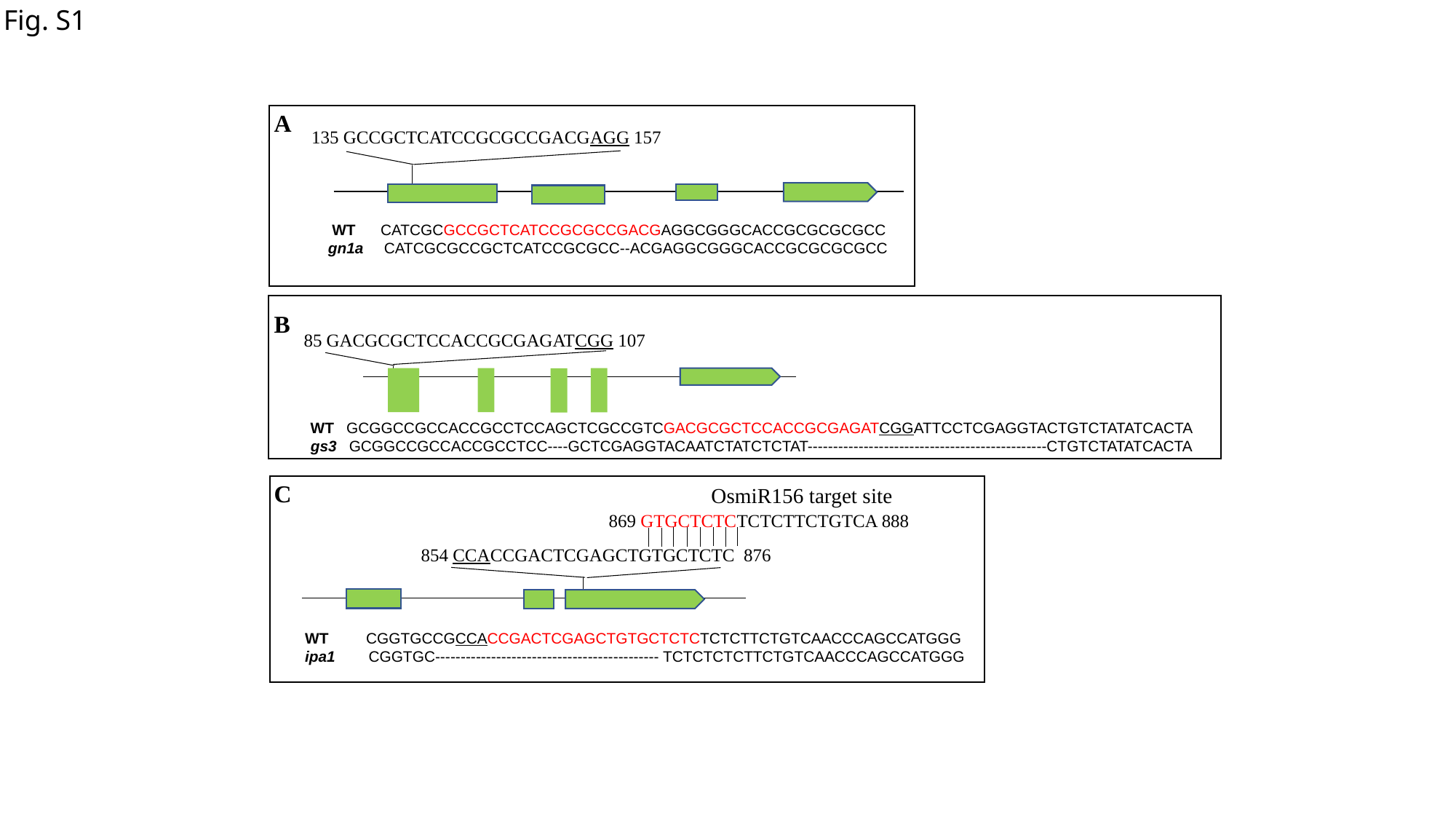

Fig. S1
A
135 GCCGCTCATCCGCGCCGACGAGG 157
 WT CATCGCGCCGCTCATCCGCGCCGACGAGGCGGGCACCGCGCGCGCC
 gn1a CATCGCGCCGCTCATCCGCGCC--ACGAGGCGGGCACCGCGCGCGCC
B
85 GACGCGCTCCACCGCGAGATCGG 107
WT GCGGCCGCCACCGCCTCCAGCTCGCCGTCGACGCGCTCCACCGCGAGATCGGATTCCTCGAGGTACTGTCTATATCACTA
gs3 GCGGCCGCCACCGCCTCC----GCTCGAGGTACAATCTATCTCTAT-----------------------------------------------CTGTCTATATCACTA
C
OsmiR156 target site
869 GTGCTCTCTCTCTTCTGTCA 888
854 CCACCGACTCGAGCTGTGCTCTC 876
WT CGGTGCCGCCACCGACTCGAGCTGTGCTCTCTCTCTTCTGTCAACCCAGCCATGGG
ipa1 CGGTGC-------------------------------------------- TCTCTCTCTTCTGTCAACCCAGCCATGGG

## Slide 2
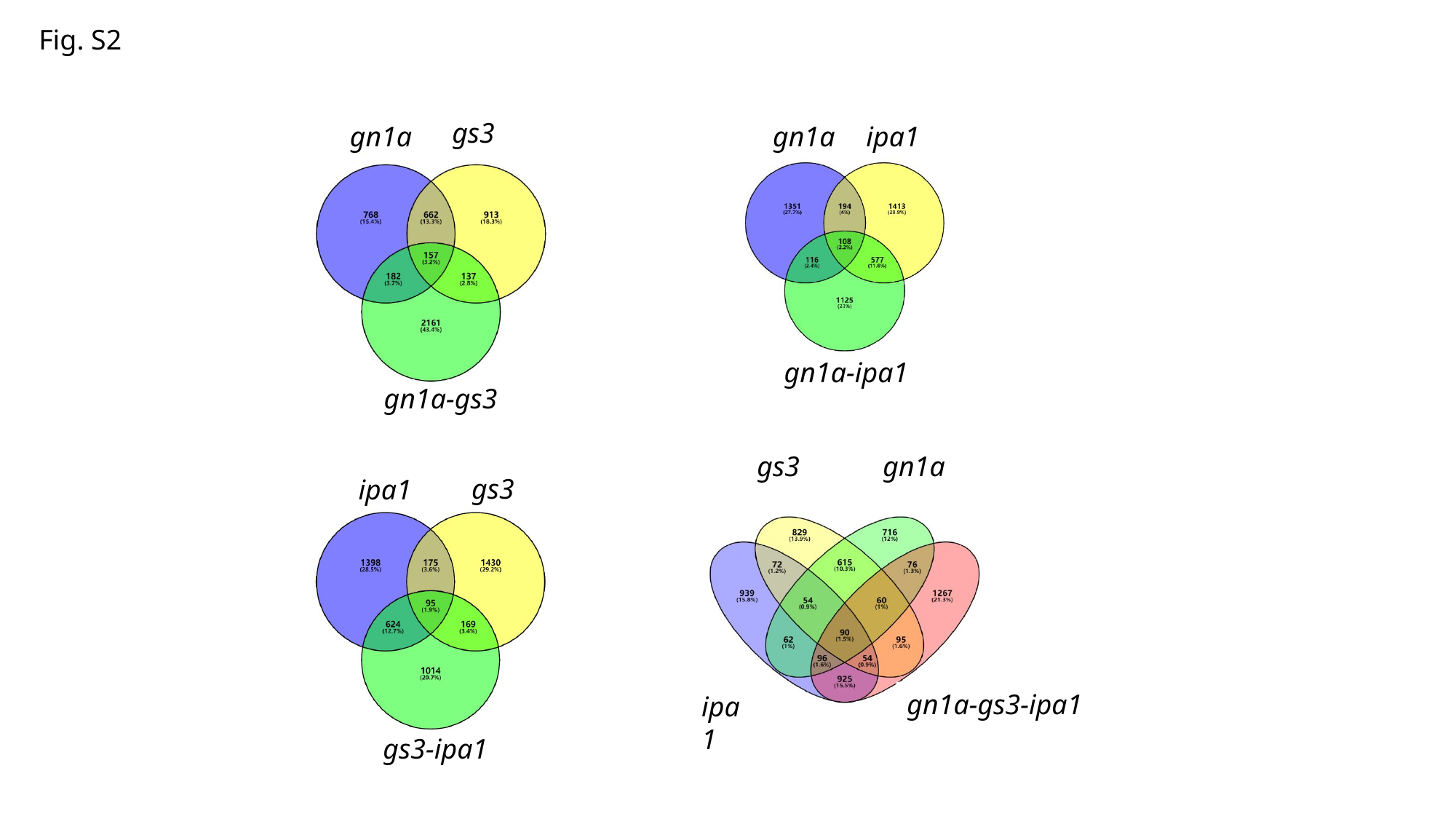

Fig. S2
gs3
gn1a
gn1a
ipa1
gn1a-ipa1
gn1a-gs3
gs3
gn1a
gs3
ipa1
gn1a-gs3-ipa1
ipa1
gs3-ipa1
